# Behavioral observations on the White-breasted Thrasher (*Ramphocinclus brachyurus brachyurus*): conservation implications

**DOI:** 10.1007/s10211-014-0207-3

**Published:** 2014-10-22

**Authors:** Jean-Raphael Gros-Desormeaux, Thierry Lesales, Alexis-Georges Tayalay

**Affiliations:** 1 Centre National de la Recherche Scientifique (CNRS), UMR 8053 (Centre de Recherche sur les Pouvoirs Locaux dans la Caraïbe), Institut Ecologie et Environnement (InEE), Université des Antilles et de la Guyane, BP 7209, 97275 Schoelcher Cedex, Martinique France; 2 Cabinet d’Ingénierie Homme Et Nature Conseil Environnemental (CIHENCE sarl), 112 Avenue de Paris CS 60002, 94306 Vincennes Cedex, France; 3 Association Ornithologique de la Martinique (AOMA), 59, Route de Pointe Fort, 97231 Le Robert, Martinique France

**Keywords:** White-breasted Thrasher, Behavior, Ecology, Martinique, Predation, Conservation

## Abstract

The White-breasted Thrasher (*Ramphocinclus brachyurus brachyurus*) is surviving at the tip of the Caravelle peninsula in Martinique, on a 5 km^2^ territory. Once widespread throughout the island, this passerine was on the verge of extinction in the 1950s but managed to recover. The creation of the Caravelle Nature Reserve in 1976 contributed to the protection of its habitat, but little is known about the factors behind the slow population growth registered in the past decades. A year-long ethological study was launched by the Regional Natural Park of Martinique (PNRM) in order to understand the status of this endangered species. In spite of some limitations, original observations shed new light on the behavior of this endemic species. New calls and a song were identified for the White-breasted Thrasher. The study highlights seasonal variations in the bird’s feeding behaviors and some behavioral plasticity in its reproductive strategies. Individuals appear to be exposed to strong predation pressure, especially during the breeding season. The confirmation of the modus operandi of rats against White-breasted Thrashers’ nests should help improve the conservation policy of this bird.

## Introduction

With about 12,500 vertebrate and plant species, the Caribbean Islands Hotspot is one of the world’s greatest centers for biodiversity and endemism. Five hundred sixty-four birds are present, of which 26 % are endemic (CEPF 2009). One of them, the White-breasted Thrasher (*Ramphocinclus brachyurus*), is found only in the Lesser Antilles islands Martinique and St. Lucia. With a total population estimated at 1,900 individuals and given its highly restricted range, it is ranked as “endangered” (IUCN 2012). Recent and ongoing studies on the declining population of the St. Lucia White-breasted Thrasher (*Ramphocinclus brachyurus sanctaeluciae*) have greatly improved the scientific knowledge of the behavior and the threats that this species faces. Evidence of cooperative breeding, philopatric tendencies, and strong vulnerability to habitat deforestation has been identified (Temple 2005; Temple et al. 2006; Morton 2009; White 2009; Young et al. 2010). However, the Martinique White-Breasted Thrasher appears to be less known than the St. Lucia subspecies (*R. b. sanctaeluciae*).

**Fig. 1:**
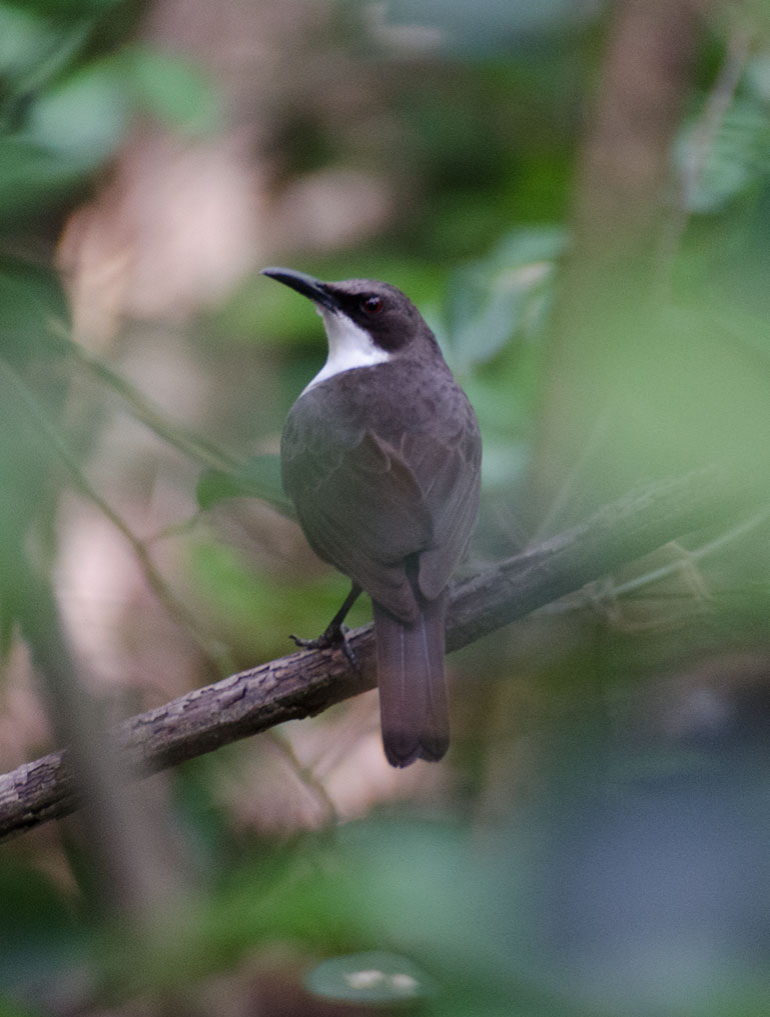
Martinique White-breasted Thrasher (*Ramphocinclus brachyurus brachyurus*)

The subspecies found in Martinique (Fig. 1) was declared on the verge of extinction in 1905, then considered extinct in 1950, but managed to survive and seems to be slowly increasing its population in spite of adverse conditions. Described as widespread on the island in 1876, sighted between Trois-Ilets and Saint-Pierre, the species was already believed to be on the brink of extinction in June 1905, at the Fourth International Congress of Ornithology, which was held in London. Rosthschild (1907) attributes the virtual disappearance of the species from Martinique to the eruption of Mount Pelée in 1902. Though, the introduction of mongooses (*Herpestes javanicus*) to Martinique in 1890 (according to Pinchon 1967) coincides with the sharp decline. Thus, the eruption of Mount Pelée may have worsened an already delicate situation, given the changes in the environment by deforestation and the introduction of predators (e.g., rats *Rattus rattus*, mongooses, and cats *Felis silvestris catus*). In 1950, American ornithologist James Bond, in his catalog of the birds of the Caribbean, considered the *R. b. brachyurus* extinct in Martinique. However, in 1951, the capture of an individual in the area of the lighthouse on the Caravelle revealed that the White-breasted Thrasher was still surviving on the peninsula but was extremely rare (Pinchon and Bon Saint Come 1951). Bond (1966) reported that two individuals were observed in the same region. In 1976, the Caravelle Nature Reserve was established. Since then, it appears that the population of White-breasted Thrashers has increased significantly over the decades. Ornithologists counted 15 pairs in 1987 (Benito-Espinal and Hautcastel 1988), while Evans (1990) noted 40 pairs. The AEVA studies in 1994, and between 1995 and 1996, confirmed the presence of 40 pairs in the reserve. The latest population estimates of *R. b. brachyurus* in 2005 established that the number of individuals should be between 200 and 400 over an area of 5 km^2^ of the peninsula (AOMA 2008).

Based on the high vulnerability of the Martinique subspecies (class. IUCN), it was necessary to set conservation actions from an empirical approach that can challenge the traditional ecological knowledge and further formulate new hypothesis. We currently have few elements that would help to understand the observed shift in population dynamics. Data on the White-Breasted Thrasher’s behavior and ecology before 1990 are very limited; we rely mostly on descriptions by 19th century naturalists and on some information collected by Pinchon (1967). Our study was conducted between November 2011 and September 2012 and aimed at gathering basic behavioral data on this little-known species. Our observations on territoriality, breeding biology, social interactions, and foraging behavior may contribute to a better understanding of the needs of this species and to justify adapted conservation actions (Sutherland 1998).

## Materials and methods

### Study site

**Fig. 2:**
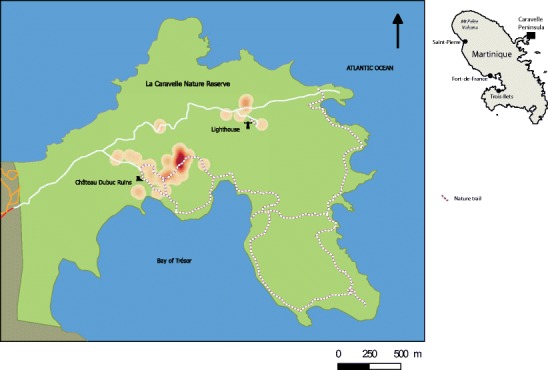
Locations of nesting and foraging observations of the Martinique White-breasted Thrasher (*Ramphocinclus brachyurus brachyurus*)

The Caravelle peninsula is a small peninsula in the northeast of Martinique (Fig. 2). It extends from east to west over a distance of about 10 km, with a width varying from 1–4 km. At the end of the 17th century, the Caravelle forest underwent significant deforestation for agricultural purposes. Abandoned at the end of the 18th century, the area devoted to farming had decreased significantly in favor of savannas, thickets, and dry secondary forests. From the 1970s, a series of measures led to the establishment of a nature reserve on the eastern tip of the peninsula. The Caravelle Nature Reserve, one of the driest areas in Martinique, is 3.8 km^2^ divided into various topographic units: rolling hills, small ravines, bays, coves, and cliffs. Its highest point is estimated at 148 m.

### Meteorological data

On average, total annual rainfall is less than 1,000 mm on La Caravelle Peninsula. During the dry season (February to April), precipitation is only 14 % of the annual total rainfall compared to 37 % during the rainy season (August to October). On average, daily temperatures are usually strictly above 25 °C. From October 2011 to August 2012, meteorological data available from Météo France and the General Council of Martinique highlight some remarkable anomalies during the period of observation (Table 1). The year 2011 was characterized by heavy rainfall, outranging the 1947 record of annual rainfall. The year 2012 was, however, distinguished by an early start of the dry season in the month of January with large deficits of rain. Meteorologists have also noted that the trade wind regime was stronger than usual, and the dry season was ranked as one of the four windiest in the last 50 years. Global rainfall deficits are still mitigated by some exceptional episodes of intensity. Paradoxically, May 2012 was particularly rainy and appears to be one of the five wettest months of May since 1947, recording a 147 % surplus. The months of June and July were once again deficient, while excess rainfall events were recorded in August. The temperatures were generally warmer than seasonal norms, with minimum temperatures higher than average. This is part of a general trend of rising temperatures noted over the past decade in Martinique. August 2012 was marked by the passage of Tropical Storm Ernesto in the Lesser Antilles. This episode generated strong wind gusts; 107 km/h winds were recorded on the Caravelle peninsula, on August 3, 2012.

**Table 1:** Total monthly rainfall from October 2011–August 2012

	October	November	December	January	February	March	April	May	June	July	August
Rainfall (mm)	191.2	221.2	237.8	34	68.2	117.6	145.6	427.6	58.2	128.6	262.2

Source: General Council of Martinique

### Habitat description

The soils of the Caravelle Nature Reserve are fairly new and rudimentary and are particularly vulnerable to erosion. However, thanks to the vegetation, leaf litter and plant debris allow the thickening of a horizon of arable land. Erosion helps create biologically rich environments due to sediment transport by runoff and accumulation of generated alluvial deposits downslope. Coastal environments are split into three main types: mangroves, beaches, and cliffs. Inland, we must distinguish herbaceous savannas, thickets, and sylvatic formations. Savannas are completely open environments, and they are scattered throughout the nature reserve, both inland and along the coast. Dense thickets and closed shrub lands develop on upper slopes where the soils are shallow. The architecture of sylvatic formations varies depending on the topography. The forested areas on the slopes are more open than the biotopes of alluvial basins. The latter have a greater plant biomass (i.e., soils are deeper and more humid), and litter decomposition is more pronounced. *R. b. brachyurus* is present only in some specific sites of the Caravelle peninsula. The bird is found in wood areas on slopes and in alluvial basins. This species is highly specialized and reliant on this type of habitat.

### Data collection

Extensive literature search was necessary to collect the scientific information about the two territories where the *R. brachyurus* is present, namely Martinique (Caravelle Peninsula) and St. Lucia (Northeastern coast). This phase was organized by consulting general and specialized bibliographic databases. We relied mainly on the Caribbean collections from the University of the French Antilles and on the Biodiversity Heritage Library database. Local naturalists and scientists also provided rare insights through interviews and unpublished reports. In addition, open interviews with the employees of the Caravelle Nature Reserve and some inhabitants of the peninsula allowed us to collect empirical ecological knowledge on the *R. b. brachyurus.*

**Fig. 3:**
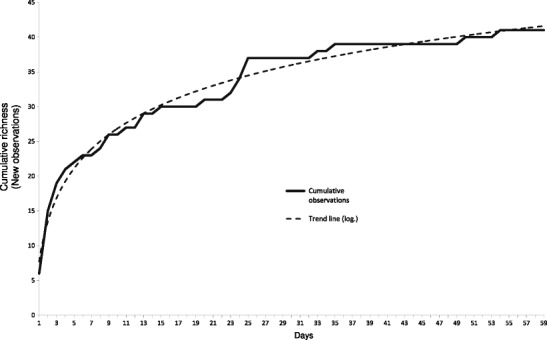
Graph of the cumulative functional units richness

In Martinique, we carried out fieldwork between November 2011 and September 2012 in the Nature Reserve (Fig. 2). During 167 sequences of at least 6 h of observation (half days), we witnessed the entire activity cycle of the White-breasted Thrashers, from sunrise to sunset. The distribution of observations was 31 half days from November to January, 65 half days from February to April, and 71 half days from May to September. The data collected using the direct observations protocol is divided into 59 actual dates of observation, which raised 2,537 descriptions of elementary behavioral (or functional) units of the White-breasted Thrasher. The functional units are basic actions and characteristics of the behavior. Each observation was described by simple action verbs: to fly, to follow, to call, to scratch, to smooth, to perch, to give, etc. They are used to define the behavior in an objective manner, limiting biased interpretation. The sample was evaluated based on an adaptation of the Monte Carlo method (Ferry and Frochot 1970; Blondel 1975; Vanpeene-Bruhier et al. 1998). It is calculated from the last point *S n* and the penultimate *S n* − 1 by the equation: *S n* = S *n* − 1 − *a*/*N*, where *a* is the number of observations of frequency 1 and *N* is the total number of observations. Assessing the sampling pressure, the slope of the curve (Fig. 3) shows the deficit needed to acquire the total set of elementary behavioral units. The curve flattens considerably from a dozen half-days of observation, where the ratio (*a*/*N*) is about 0.016.

For this study, we applied a continuous sampling approach so that we could record as many auditory and visual observations as possible (Martin and Bateson 2007). Two observation techniques were implemented: on the lookout from a fixed point and the exploration itinerary. Both approaches require the implementation of protocols to limit disturbance. The movements should be slow and silent and the color of clothing suitable for vegetation. Camouflage nets were also used in some lookout situations. When an individual was detected visually, the observation was logged using a basic digital recorder (Olympus VN8700PC). The bird vocalizations were also recorded through the built-in microphone (frequency response 70 Hz–19 kHz).

While collecting behavioral data, each observation area was identified through ecological data specifying the sampling conditions: the date, time, geodetic coordinates, temperature and humidity, leaf litter temperature, luminosity, wind speed, and specific comments related to the state of the weather. Field logs were integrated into an information system, which consisted of entities such as behavior, eco-ethology, and station. The behavioral table includes the observations that are transcribed in the form of elementary units of behavior. The eco-ethological table transcribes observations on predators, interspecific and intraspecific interactions, and disturbances. The station table contains all information on sampling conditions.

In order to limit the disturbances during the critical breeding season, the observation techniques described above were completed with the installation of automated camera traps around nests. We used motion-triggered cameras with IR night vision capabilities (Ltl 512A, 12MP); when activated, they were set to take a picture and a 1-min video clip. They were installed within 2–5 m of the nests and strapped on a tree. The automated observations allowed useful behavioral observations within the nest, day and night; this enabled us to gather an additional 148 days of indirect observations. The data collected using this technique were transcribed into the eco-ethological table. Capture and banding were not allowed in the context of this study.

A Pettitt homogeneity test (1979) was applied to the time series of each elementary unit of action. This test determines whether the observations series are homogeneous over time or whether there is a moment when a significant shift occurs. The acoustic elementary units of behavior are represented by sonograms obtained through the Audacity digital audio editor software.

## Results

**Fig. 4:**
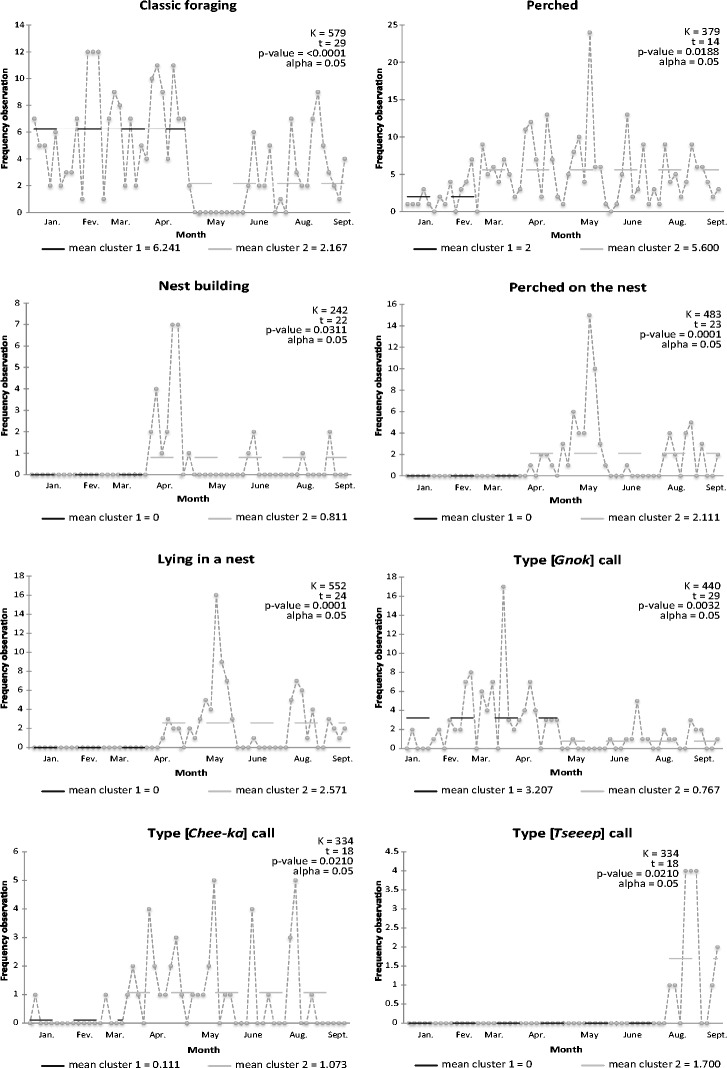
Results for a homogeneity test of Pettitt for White-breasted Thrasher’s (*Ramphocinclus brachyurus brachyurus*) behaviors

The homogeneity test conducted in the time series of each elementary unit of action from the behavior information system found a significant variation in the analysis of the following time series: classic foraging; perched; foraging and singing; trembling wings, shaking, and pecking a leaf or pecking a twig; nest building; perched on the nest; lying in a nest; type [*Gnok*] call; type [*Chee-ka*] call; complex call type; and type [*Tseeep*] call (Fig. 4). Significant changes in the behavior were observed during the April 2012 fieldwork. The following time series highlight a strong shift (increase or decrease) of their observation frequencies during the May 2012 fieldwork campaign: classic foraging; perched; foraging and singing; trembling wings; nest building; perched on the nest; lying in the nest; and type [*Gnok*] call, type [*Chee-ka*] call, and complex call type. Three particularities are worth noting: (1) a regular increasing tendency for sightings of perched White-breasted Thrashers since February 2012, (2) a remarkable increase of nest building in April, then in June and later in August 2012, and (3) the first type [*Tseeep*] calls, in August 2012.

The *R. b. brachyurus* was often observed engaging in behaviors related to feeding on leaf litter on the ground [frequency 80 %; *n* = 246]. Birds are seen moving, walking, and hopping, and they scan the ground in a squatting posture, lifting, and tossing the leaves with their beak. They can sometimes pursue prey rapidly, and they peck frequently. The White-breasted Thrasher can also dig the soil with its beak. This activity leaves characteristic scraping traces on the ground. During this activity, they raise the head regularly to observe their environment.

**Fig. 5:**
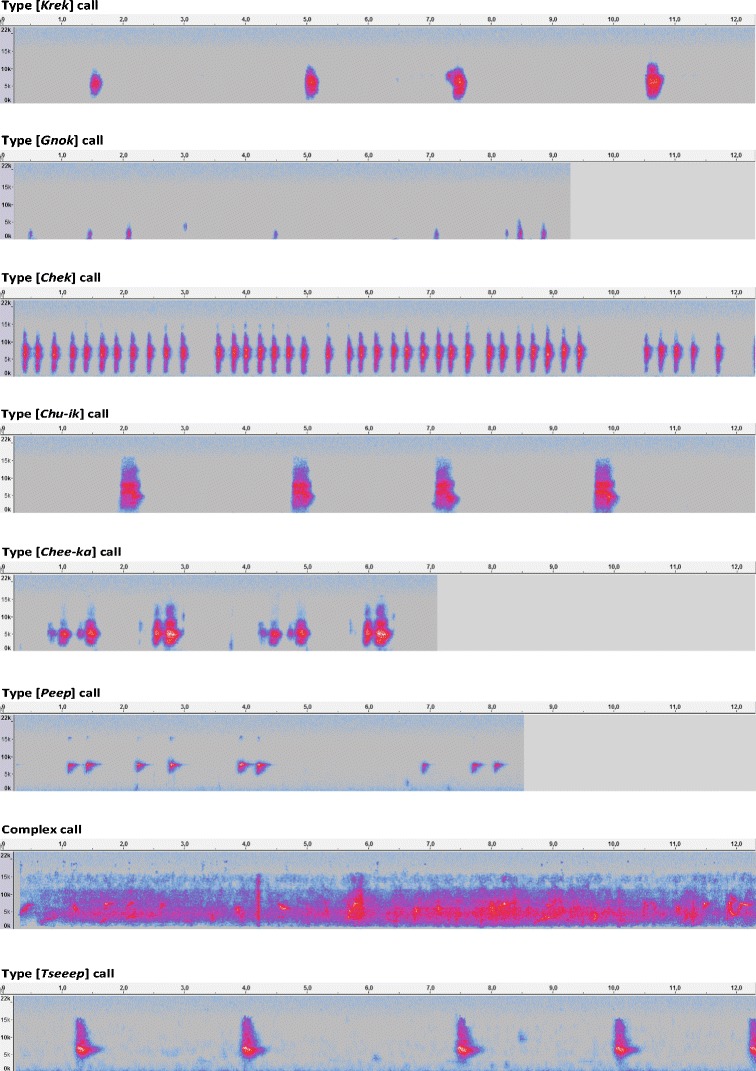
Sonograms of White-breasted Thrasher’s (*Ramphocinclus brachyurus brachyurus*) vocal repertoire (created with Audacity)

The *R. b. brachyurus* were heard and observed emitting several types of very characteristic calls (Fig. 5). The type [*Krek*] call is monosyllabic and metallic, which can be isolated or repeated [*n* = 255]. The type [*Gnok*] call is deep and monosyllabic, issued at various intervals [*n* = 116]. This type of call seems to occur when an individual on the ground is seemingly intrigued by a gradual change in its environment. We describe this as an early warning, or pre-alert call, because individuals do not necessarily interrupt their activity. The type [*Chek*] call is hoarse and monosyllabic, repeated depending on the degree of individuals’ excitement [*n* = 138]. This cry is emitted when a threat has been detected in the surrounding environment. The type [*Chu-ik*] call is bi-syllabic, which can be isolated or repeated [*n* = 84]. It is often preceded by the type [*Krek*] call. The type [*Chee-ka*] call is bi-syllabic, issued by individuals in flight [*n* = 46]. The type [*Peep*] call is high and monosyllabic repeated several times [*n* = 83]. The only song identified for the *R. b. brachyurus* is a complex call composed of chirps and trills [*n* = 22]. Chicks that had left the nest emit shrill, repeated monosyllabic calls: [*Tseeep*]. Slight variations of those calls were heard sometimes. These events are rare and could be attributed to juveniles; therefore, they were not included in this typology. Other guttural sounds, sort of clucking and chirping both low and complex, were observed around nests. They seem to relate to exchanges either between adults or between adults and chicks in or around the nest.

Personal observations, interviews, and literature search highlight a number of non-quantifiable observations. These results help define some traits regarding interspecific and intraspecific interactions and reactions to disturbances. Five potential predators of the White-breasted Thrasher were regularly observed on the Caravelle Nature Reserve. The mongoose, the Broad-winged Hawk (*Buteo platypterus*), the common opossum (*Didelphis marsupialis*), the domestic cat, and the rat were sighted on White-breasted Thrasher’s territories. The *R. b. brachyurus* displays a more aggressive behavior during its breeding season, but it can flee in the presence of Carib grackles (*Quiscalus lugubris*) and breeding hummingbirds (*Orthorynchus cristatus*) (Table 2). It is worth noting that alert calls led the chicks to hide in the nest (Table 3). The three main causes of disturbance observed triggered reactions ranging from alert calls and flight to more serious responses including the destruction of a nest (Table 4).

**Table 2:** Interspecific interactions between the White-breasted Thrasher (*Ramphocinclus brachyurus brachyurus*) and other species at Caravelle Nature Reserve

Species	No nesting activity	Nesting activity
*Saltator albicollis*	Take flight	Attack
*Quiscalus lugubris*	Take flight	No observation
*Turdus nudigenis*	No observation	Attack
*Icterus bonana*	Type [*Chek*] call	Attack
*Coccyzus minor*	Type [*Gnok*] call	Attack
*Orthorhyncus cristatus*	Take flight	No observation
*Herpestes javanicus*	Type [*Chek*] call	Attack

**Table 3:** Intraspecific interactions: the White-breasted Thrasher (*Ramphocinclus brachyurus brachyurus*)

Behavior	Comment
Follow flying	To follow with or without aggression
Parading	Take flight, jump on the other, neck stretched, observing neck stretched, trembling wings
Hiding	Chicks shrink back in the nest after type [*Chek*] call of adults
Feeding the incubating individual and chicks	To supply the incubating individual and chicks with berries and/or invertebrates

**Table 4:** Disturbances and their effects on the White-breasted Thrasher (*Ramphocinclus brachyurus brachyurus*)

Disturbance	Consequences
Loud hikers	Sudden flight; type [Chek] call; type [Gnok] call; type [Chek] call and take flight; type [Gnok] call and take flight
Passage of a low flying helicopter	Type [*Chek*] call
Trail maintenance	Nest missing

The list of fauna inventoried in the leaf litter at observation stations included *Hymenoptera* (ants), *Coleoptera* (Elateridae larvae, bark beetles, grubs), *Dermaptera* (earwigs), *Diptera* (phorid flies), *Thysanoptera* (thrips), *Orthoptera* (crickets), *Hemiptera* (larvae), *Lepidoptera* (caterpillars), *Heteroptera* (*typical bugs*), arachnids (spiders, pseudoscorpions), gastropods (snails), *Hylodes*, and Vincent’s least gecko (*Sphaerodactylus vincenti*). The White-breasted Thrasher had previously been observed pecking a clove of fresh seeds from the limber caper (*Capparis flexuosa*) and the red fruit of the *Paullinia cururu*. This observation was made in the month of August, during a severe drought (AEVA 1994). Although frequently foraging in the undergrowth, individuals have been sighted foraging in open spaces on small paths. During the first phase of nesting, and until eggs hatch, we found that the *R. b. brachyurus* fed mainly on small fruits, such as berries from the Guadeloupe marlberry (*Ardisia obovata*) and lathberry (*Eugenia cordata*).

Layings and broodings were observed on 15 different sites between May and August. Several studies have already been carried out on the nesting supports used by White-breasted Thrashers (AEVA 1994; Béranger 2007; AOMA 2008). Among the shrub species, the red rodwood (*Myrcia citrifolia*), the lathberry (*E. cordata*), the mapou (*Pisonia fragrans*), and the Guadeloupe marlberry (*A. obovata*) were most frequently used for building nests. Nests were then entangled in the uppermost fork between 1.5 and 5 m. The *R. b. brachyurus* can also use more complex materials, installing its nests in more developed shrubs; in this case, the nest is built in the fork of horizontal branches. A pair builds its nest within 4–5 days, alternating construction activities and foraging.

Once the nest is complete, the female lays one to two blue eggs, and a single individual incubates for 12–14 days. The incubating adult leaves the nest regularly, usually for food, but rarely spends more than 15 min out of the nest. The other partner remains in the immediate vicinity of the nest, perched or foraging, sometimes helping to supply the incubating individual with berries and/or invertebrates. The partner that remains outside the nest is aggressive in chasing birds that pass nearby. Bénito-Espinal and Hautcastel (2003) and AOMA (2008) have also reported this aggressive nesting behavior. The former reports that the pair can break the eggs and abandon the nest if they are disturbed. At the time of hatching, the two partners are seen perched on the edge of the nest. The altricial chicks are blind and without fluff at the time of hatching. Pieces of eggshells are evacuated and transported away from the nest, as well as white fecal sacs produced by the chicks. The nest and its surroundings, therefore, remain very clean. The parents take turns about every 5 min, feeding the chicks with invertebrates. At the end of the first week of life, the chicks become more active, and they can be seen stretching, lifting their heads out of the nest, and flapping their wings. The chicks fledge 11–12 days after hatching. They cannot yet fly and therefore fall to the ground. Adults continue to feed them when they are perched in shrubs or on the ground. Chicks have an evenly brown plumage, and their iris is dark brown, which then changes to a clearer color. In the second month, after the first molt, they can gradually acquire a white plumage on the chest and throat.

Five different types of situations were noted: brooding observations but no information on the reproductive success, hatching observation with chicks in the nest, observations of chicks leaving the nest, brooding observations with evidences of predation, and brooding observations with proven predation. In case of failure, the pair can start a new reproductive cycle very quickly, 5 days after an episode of predation, and individuals were observed in the same territory, taking the twigs from the predated nest and building another nest about 10 m away.

Banding campaigns conducted in the 1990s by AEVA (1996) gave some results regarding dispersal behaviors of the White-breasted Thrashers. Eleven banded Thrashers were recaptured in 1994 and 1995, only three individuals had traveled distances between 400 and 1,000 m, five had settled in the same area of their first capture, and three had traveled a distance between 150 and 250 meters (AEVA 1996).

## Discussion

Although the assessment of the sampling pressure proves satisfactory (*a*/*N* = 0.016), field observations have often been partial especially given the configuration of the undergrowth in which the White-breasted Thrasher inhabits. Dense vegetation often masks individuals who frequently move on the ground or in the tree-shrub strata. These conditions also make it difficult to avoid creating a disturbance in the undergrowth while walking on the dry leaf litter. The lack of sexual dimorphism does not allow the differentiation between individuals and sexes. This complicates the understanding of the species’ social, territorial, and reproductive behavior. For example, when monitoring nests, it was often impossible to determine which individual was responsible for brooding and whether the couple took turns or not. Finally, the duration of this ethological study over a period of 11 months does not completely confirm some of the observed behaviors. Parts of the field observations limited to a season may be only short-term, probably depending on specific environmental variables (e.g., weather and fruiting phenology). From the data collected, we attempt to define and describe the different behaviors of the species, based on environmental conditions and the objectives sought by individuals. However, in the absence of formal determination of sex and age of the individuals involved in the observation sequences, we cannot attempt to make absolute interpretations of social ties and territorial characteristics of the species.

Throughout our observations, we noted no obvious effects of climate anomalies on the individuals of White-breasted Thrashers. Heavy rainfall and consequent runoff in May washed the leaf litter, but the birds had already changed their diet and were no longer foraging on the ground at this time. However, a cross-analysis of the rain distribution (Table 1) and of the two elementary units of behavior “perched” and “foraging” suggests a close correlation (Fig. 4), which demonstrates an impact of the rain on the White-breasted Thrashers behavior. In spite of the rains which bent some nest supports, monitored active nests remained in place without any damage to the nestlings. One exception was a nest that was under construction and was later found degraded and abandoned. Two old nests were damaged and knocked down during the rainy periods. Wind gusts generated by tropical storm Ernesto on August 3 2012 broke many small branches and leaves at the exposed slopes of the nature reserve. Several nests were found on the ground, but none of the monitored White-breasted Thrasher nest suffered serious damage. It is likely that non-monitored nests were destroyed by these two climatic events. Peak activities related to nesting—nest building, perched on the nest, and lying in a nest—noted in June and in August (Fig. 4) may also be related to the heavy weather conditions.

Based on traditional ecological knowledge (Huntington 2000), only three calls were attributed to the *R. b. brachyurus*; however, this study identified new calls and a new song. The call is distinguished by its simplicity, namely its brevity and structure built from one or a few syllables. In contrast, the song is characterized by long and complex vocalizations. The identification of new vocalizations for the White-breasted Thrasher should help improve detection, which could lead to better monitoring and protection of the species.

The *R. brachyurus* is a territorial bird that generally lives in groups composed of up to five individuals (Temple 2005). It appears that the *R. b. brachyurus* are permanently spread over the territories, which they occupy in pairs or groups of three to five individuals. The structure of these territories is most obvious during the breeding season, when individuals can be seen in pairs around nests. Observations also lead us to speculate about the existence of buffer zones beyond permanent territories. Those areas would be used to increase access to food resources, especially when conditions are favorable. On the peninsula, the variation of vegetation during the dry season appears to play a limiting role in some areas. In addition, during the reproductive phase, we observed birds as pairs, and groups of White-breasted Thrashers were no longer observed. Individuals, probably young adults, dispersed from their native territories and occupied peripheral areas where they did not reproduce.

Social relations of the White-breasted Thrashers seem varied and subtle. Postures of the species with an outstretched neck, wings drooped or held apart and/or shaken can be associated with displays. They could be demonstrations of distrust between individuals, which may be related to defense mechanism. There were several instances when a pair of White-breasted Thrashers was seen perched, with their wings agitated, and neck stretched in the direction of a third individual, which was also perched with an outstretched neck, followed by the pair, or one member of the pair flying in pursuit of the intruder. This posture was also observed among members of the same group, probably in the context of dominance. The evolution of the birds in pairs or in groups appears to be a useful anti-predatory strategy. While foraging on the ground, individuals are vulnerable to mongooses, and they can warn each other when a threat is perceived. Types [*Gnok*] call and [*Chek*] calls seem to be warning and alarm calls, and the birds can then react accordingly. Mongooses can, for instance, be harassed by several *R. b. brachyurus* flying overhead and emitting the type [*Chek*] call, until the predator leaves the territory. The song identified for the White-breasted Thrasher was always observed in thickets when several individuals were engaged in a foraging activity on the ground. It may have some social purpose; however, to date, we do not have a conclusive interpretation.

Studies and surveys generally confirm that the *R. b. brachyurus* has a varied diet. Satisfying nutritional needs seems to occupy much of the individuals’ activities during the day. If this species forages mainly on the leaf litter fauna, it can also prey on insects hiding in dead stumps or on shrubs and trees. This species has also been observed eating berries. They can sometimes regurgitate a whole berry, keeping it in the beak for feeding on it later and spitting out the seeds. This change in foraging behavior may be related to the unusually high rainfall recorded during the dry season in 2012. However, samples of the leaf litter taken at the end of April, a period marked by frequent rainfall, showed that leaf litter fauna was abundant in the traditional foraging areas. The typology of catches appeared richer than the results of previous campaigns conducted in August 1994 (AEVA 1994). The change of foraging behavior could therefore be an opportunistic strategy for the White-breasted Thrasher to preserve a vital resource to feed the chicks. The *R. b. brachyurus* feeding habits seem to show adaptation to its environment and the implementation of a survival strategy that allows it to use all available resources based on seasonal variations. In the definition of a conservation policy, we must consider the limits of this strategy in severe climatic stress, in an environment where access to water can be limited.

Reproduction is a crucial phase for the preservation of the species. The reproductive strategies should therefore help to effectively ensure a new generation of individuals. Breeding season seems to have started in mid-April with the first peak activities related to nesting. This phase was marked by the observation of individuals in search of twigs and leaves, which were carried in the beak. In the territories where previously groups of White-breasted Thrashers had been seen, only pairs remained. Social and territorial behaviors also appeared to occur with more frequent displays during this period. Two individuals carried out nest building, taking turns in all related tasks: both *R. b. brachyurus* lying in the nest, in turns, to adjust and settle the leaves and roots that make up the inside of the nests. The pairs seem to be able to build several nests sequentially or concomitantly before actively using a nest. In general, the species uses isolated bushes or shrubs, with a relatively straight and slender small-diameter rod. Seeking the flexibility of the nest support, White-breasted Thrashers seem to adopt a comprehensive anti-predatory strategy that allows them to be alerted when an attempt is made to access the nest (Tayalay 2013). The presence of old nests in the vicinity of active nests reveals that the species probably uses a particularly favorable territory for the successive construction of nests. Old nests may serve as a reserve for twigs or sometimes may be directly reused after being consolidated. Pinchon (1967) reported that the average clutch size for the *R. b. brachyurus* was three eggs. However, our field observations and interviews reveal that the number of eggs laid ranges between 1 and 2. This variation may be the result of behavioral plasticity as a response to pressure from predation. Clutch size reduction is a common reproductive strategy adopted by passerine species facing high risk of predation (Fontaine and Martin 2006).

Throughout the rearing of chicks in the nest, White-breasted Thrashers pay special attention to keeping the nest clean, removing egg shells, and fecal sacs. This is probably an anti-predatory strategy that can help limit the detection of nests. Chicks in the nest seem very inconspicuous. When a threat is perceived, including the warning call of adults, they shrink back in the nest. From April on, the scarcity of audible manifestations points at the adoption of a more inconspicuous behavior by *R. b. brachyurus* during the breeding season (Fig. 4). Once on the ground, homochromy and immobility would provide temporary protection on the leaf litter. However, partial observations tend to show that the chicks do not stay on the ground for very long, but they take refuge hopping in the bushes. They seem to resume frequenting the leaf litter once they have mastered flight.

**Fig. 6:**
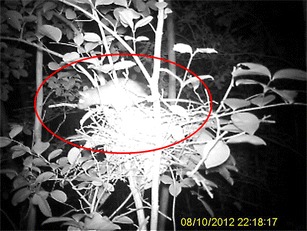
Predation of a White-breasted Thrasher’s (*Ramphocinclus brachyurus brachyurus*) nest by a rat (*Rattus rattus*)

The rate of nesting failure seems rather high; the first evidence of chicks hopping and squeaking on the ground was recorded in August, nearly 4 months after the first peak of nesting activities. The main cause of nest failure is the predation of eggs. We had strong suspicions of predation on four nests found empty during the breeding season, and camera traps helped to identify the predators. Rats entered the nests at night and fed on the eggs (Fig. 6). We found that predated nests were particularly vulnerable because they were accessible by branches in contact with their support. We also suspected predation in the case of chicks that disappeared in three of the territories that we monitored. They were observed leaving the nest but were never seen again. When on the ground, the chicks are particularly vulnerable to mongooses. The breeding season continued until the end of September, and we witnessed up to four nesting cycles; i.e., pairs of *R. b. brachyurus* attempted to produce up to four successive broods. However, the increase in the number of broods is not necessarily related to failure. We observed a brooding individual, as well as an adult caring for a chick on the ground, while in the same territory. The adult appeared to be accompanied by a third adult who contributed to feeding. This may be a case of cooperative breeding already described and studied by Helen Temple in St. Lucia (2005). The White-breasted Thrasher appears to apply strategies that seem particularly pugnacious to ensure reproductive success. However, despite their considerable efforts from April to September, reproduction results were low and late: the juvenile call [*Tseeep*] type was not heard before August (Fig. 4). The number of juveniles observed seemed low after 4 months of breeding activity. These data need to be supplemented in the future with regular monitoring during this critical phase. The identification of predators and characterization of its mode of operation are important steps for the conservation of the species. This would facilitate a proactive conservation policy on the Caravelle Nature Reserve.

With the use of banding, studies conducted by the AEVA (1996) and Temple (2005) have established certain features of *R. brachyurus*’ dispersal strategies. This species has strong philopatric trends; i.e., individuals prefer to inhabit areas close to their natal territory. This characteristic could explain the short dispersal of the species and therefore its limited distribution in Martinique. The *R. b. brachyurus* appears to be a rather sedentary species. However, data on their dispersal remain incomplete, and it is necessary to understand the strategies involved for estimating the potential of the species’ future dispersal. A wider distribution of White-breasted Thrashers in Martinique would likely reduce its vulnerability. Therefore, it is essential to band individuals for the consistent monitoring of this species, as well as for adding statistical analysis. The authors regret that the banding campaigns initiated in the 1990s have not been extended.

This lack of banding prevented us from collecting sufficiently detailed data for a full comparison of the behaviors of the Martinique and St. Lucia subspecies, but some differences are worth noting. Both subspecies evolved at the eastern coast of their respective islands, in dry forests where the red rodwood (*M. citrifolia*) is common. However, the *R. b. sanctaeluciae* tends to prefer dense mature woodlands, with a canopy height above 5 m, and some populations can occupy both the interfluves and the valleys of their habitat (Temple 2005). Contrary to the Martinique White-breasted Thrasher, the St. Lucia subspecies face mostly indigenous predators which attack the nests, such as the St. Lucia boa (*Constrictor orophias*), the common opossum, and the Broad-winged Hawk (Temple 2005; Morton 2009; Stephen Lesmond personal communication). The introduced mammals (rat, mongoose, and cat) are not common in the St. Lucia habitat known to be infested with the poisonous snake fer-de-lance (*Bothrops caribbaeus*). In St. Lucia, deforestation appears to be the most serious threat to the survival of the *R. b. sanctaeluciae*. Only 4 % of its territory is protected through a forest reserve (Temple 2005), whereas in Martinique, about 50 % of the *R. b. brachyurus* habitat is protected. More research is needed on the ethology of these two subspecies in order to enhance the conservation efforts.

## Conclusions

The data collected on the behavior of the White-breasted Thrasher contribute to a better understanding of the species and the challenges that it faces in Martinique. Survival strategies for the exploitation of food resources seem specifically adapted to the environment. *R. b. brachyurus* has a varied diet that allows it to feed on both animal proteins and fruits. The plasticity demonstrated in the feeding strategy of the White-breasted Thrasher emphasizes the species’ ability to withstand and adapt to adverse conditions. New research should be undertaken in order to better link fruiting phenology, weather, and the limits of the Thrasher’s opportunism, especially in the context of climate change. The *R. b brachyurus* appears to have no known competitors within the Nature Reserve, but observations of a pair of White-breasted thrashers (*R. b. brachyurus*) taking flight in the presence of Carib grackles (*Quiscalus lugibris*) raise new questions about the impact of interspecific competition on its conservation.

This species displays a large array of anti-predator strategies to ensure reproduction success. However, short dispersal distances of the White-breasted Thrasher are superimposed to the presence of predators that undermine those efforts. Predation risk seems very high, with four predator species commonly observed in the *R. b. brachyurus* territories. This study sheds new light on nest predation by rats and identifies vulnerability factors based on which the managers of the nature reserve should act to guarantee better conservation of the White-breasted Thrasher.

We recommend that a rat control program should be implemented in the nature reserve, along with the monitoring of the breeding season, in order to maximize the chances of reproduction. The implementation of nest protection techniques against predators should also be considered. The new calls and song may help to improve the monitoring of the *R. b. brachyurus* population in the future.

The choice to integrate an ethological approach in monitoring the species has produced unique data on behavioral strategies to help ensure the survival of the population. However, in the context of global warming and its projected impact on the frequency of extreme weather events, the vulnerability of this species remains high.
